# β-Naphtoflavone and Ethanol Induce Cytochrome P450 and Protect towards MPP^+^ Toxicity in Human Neuroblastoma SH-SY5Y Cells

**DOI:** 10.3390/ijms19113369

**Published:** 2018-10-28

**Authors:** Jesus Fernandez-Abascal, Mariantonia Ripullone, Aurora Valeri, Cosima Leone, Massimo Valoti

**Affiliations:** 1 Dipartimento di Scienze della Vita, Università di Siena, Via Aldo Moro 2, 53100 Siena, Italy; mariantonia.ripullone@gmail.com (M.R.); leone27@student.unisi.it (C.L.); massimo.valoti@unisi.it (M.V.); 2 Molecular Horizon srl, Via Montelino 32, Bettona, 06084 Perugia, Italy; aurora.valeri@molhorizon.it

**Keywords:** cytochrome P450 enzyme system, neurodegeneration and neuroprotection, mitochondrial localization, xenobiotic toxicity, cytochrome P450 induction, Parkinson’s disease

## Abstract

Cytochrome P450 (CYP) isozymes vary their expression depending on the brain area, the cell type, and the presence of drugs. Some isoforms are involved in detoxification and/or toxic activation of xenobiotics in central nervous system. However, their role in brain metabolism and neurodegeneration is still a subject of debate. We have studied the inducibility of CYP isozymes in human neuroblastoma SH-SY5Y cells, treated with β-naphtoflavone (β-NF) or ethanol (EtOH) as inducers, by qRT-PCR, Western blot (WB), and metabolic activity assays. Immunohistochemistry was used to localize the isoforms in mitochondria and/or endoplasmic reticulum (ER). Tetrazolium (MTT) assay was performed to study the role of CYPs during methylphenyl pyridine (MPP^+^) exposure. EtOH increased mRNA and protein levels of CYP2D6 by 73% and 60% respectively. Both β-NF and EtOH increased CYP2E1 mRNA (4- and 1.4-fold, respectively) and protein levels (64% both). The 7-ethoxycoumarin *O*-deethylation and dextromethorphan *O*-demethylation was greater in treatment samples than in controls. Furthermore, both treatments increased by 22% and 18%, respectively, the cell viability in MPP^+^-treated cells. Finally, CYP2D6 localized at mitochondria and ER. These data indicate that CYP is inducible in SH-SY5Y cells and underline this in vitro system for studying the role of CYPs in neurodegeneration.

## 1. Introduction

Cytochrome P450 (CYP) is a superfamily of isozymes involved in the metabolism of xenobiotics in liver and other extra-hepatic tissues. In the brain, CYP concentration is approximately 0.5–2% of that in liver microsomes [1]. This amount is enough for some brain-CYP isoforms to play a role in tissue- and/or cell-specific sensitivity to certain xenobiotics [2]. However, the intracellular location is still a subject of study, since they seem to target either microsomes, mitochondria, or both [3,4,5,6]. Regardless of their location, some isoforms have been proposed to play an important role in neurodegeneration due to their xenobiotic metabolizing activity [7]. Thus, the different concentrations and relative distributions of isoforms in brain, together with the sensitivity to be inducible, may contribute to the variation in therapeutic response and side-effects to drugs and xenobiotics in general [8,9]. Several environmental toxins that play a role in the pathogenesis of neurodegenerative disorders are able to damage neurons mostly driven by a CYP-dependent metabolism [10,11,12]. For example, rural residence and pesticide exposure can increase the risk of developing Parkinson’s disease (PD) [13]. The current thought is that the etiology of PD results from a combination of exposure to environmental toxins and genetic susceptibility in the regulation of bioactivation and detoxification processes for exogenous compounds [14,15]. However, there is limited knowledge of the possible mechanism(s) by which CYP-bioactivated products cause impairment of the cellular functions in dopaminergic neurons [16,17,18].

CYP2B6, 1A1, and 3A4, have not been directly related to PD, but they also have an important role in the central nervous system (CNS) as they can be regulated by several xenobiotics in both the liver and the brain. In the liver, CYP2B6 is involved in the metabolism of therapeutic drugs and other exogenous and endogenous compounds [19,20]. Additionally, CYP2B6 has been found in the frontal cortex of human brain and other brain areas of African Green monkeys [21,22]. CNS-acting drugs, such as bupropion or selegiline, can be therefore be metabolized by CYP2B6 [23,24]. The expression of this isozyme is regulated by the pregnane X receptor and the constitutive androstane receptor in the liver, but little is known about its regulation in brain [25]. CYP1A1 is an inducible isoform that can be regulated by aryl hydrocarbon receptors (AhR) [26,27]. Its expression has been related to some brain areas, yet its main role in neuronal cells is not well known [28]. At intracellular level, it has been found in mitochondria and endoplasmic reticulum (ER) [3]. Also, its presence in several areas of pig brains have suggested an important role in metabolic activity due to its inducibility by several compounds such as β-NF [9,29]. CYP3A4 is a well-known oxidative enzyme that is mostly involved in the metabolism of xenobiotics, and is the most expressed isoform in human liver [30]. Its metabolic role in the CNS is still unclear, yet its genetic regulation can be mediated by the pregnane X receptor in both the liver and brain [31,32]. Due to its wide range of metabolic activity, it has been also related with drug–drug interaction of CNS-drugs, such as co-administration of sertraline and carbamazepine [33,34].

CYP2D6 is one of the most expressed isoforms in brain cells and plays a key role in CNS homeostasis. For example, it protects neurons against 1-methyl-4-phenyl-1,2,3,6-tetrahydropyridine (MPTP) toxicity, probably by inactivating it to 4-phenyl-1,2,3,6-tetrahydropyridine [35,36]. CYP2D6 is also involved in an alternative anabolic pathway of dopamine, thus taking part in important aspects of brain health and neurotoxicity [37,38]. Recently, it has been shown that CYP2D6-polymorphism affects dopamine formation through drug interaction, and therefore, may contribute to neurodegeneration [39]. Individuals with CYP2D6-poor metabolism are related to a higher risk of PD [40]. On the contrary, smokers present a higher expression of this isoform and have lower risk of neurodegeneration of dopaminergic cells [41,42]. This isoform is also known to metabolize a wide range of CNS-acting drugs such as antidepressants, opioids, selective serotonin reuptake inhibitors, or amphetamines [43,44,45,46]. Another important isoform in the CNS is CYP2E1, which can metabolize l-deprenyl in C57BL mice but not in monkeys, either in the liver or brain [47,48,49]. Despite that its mRNA expression is lower than that observed for CYP2D6 in substantia nigra, CYP2E1 is important for many key biological processes [2,50]. It is involved in the neurodegeneration processes mediated by ethanol upon chronic exposure and its induction may be regulated by p38 and ERK1/2 pathways in neurons [51]. In the brain of PD patients, the *CYP2E1* gene seems to be less methylated compared to healthy brains, leading to a higher expression of this isoform [50]. CYP2E1 has been proposed to play a role in the development of PD due to its capability to be induced, its ability to metabolize several xenobiotics that are able to cross the blood–brain barrier, and the high level of ROS production during its metabolic reactions [52,53,54]. Moreover, CYP2E1 also present polymorphisms, where the 5’ flanking region seems to be important for the metabolism of drugs [55]. Thus, further understanding in brain CYP-metabolism can be crucial for uncovering the molecular mechanisms involved in neurodegeneration and for developing new therapeutic interventions for neurological diseases. The difficult accessibility and the lack of human dopaminergic cells from substantia nigra has underlined the neuroblastoma SH-SY5Y cell line as a useful tool for the study of PD [56]. Therefore, other groups have used this cell line for the study of many features linked to this neurodegenerative disease including the induction and protective role of CYP against toxic compounds related to PD [35,57,58]. On the other hand, there are several xenobiotics able to promote the expression of CYP(s), such as β-naphtoflavone (β-NF) and ethanol (EtOH). β-NF is the agonist of the well-known AhR, which is involved in the regulation of some CYP isoforms. Also, it has been related to a partial neuroprotection against MPTP in a mouse model of PD [59,60,61]. EtOH is the most studied compound for CYP2E1 induction in both in vitro and in vivo experiments [58].

Here, we introduce a new study where β-NF and EtOH have been used to investigate the induction of CYP isozymes in neuroblastoma SH-SY5Y cells and their intracellular localization. We found that CYP2D6 can play an important role in the metabolism of xenobiotics in this cell line.

## 2. Results

### 2.1. Induction of CYP2D6 and 2E1

Preliminary experiments were performed in order to measure the toxicity of each inducer by MTT assay. The results showed that the maximum concentration that had no effect on SH-SY5Y cell viability after 48 h of incubation were 4 µM for β-NF and 100 mM for EtOH (Figure S1). Moreover, these concentrations did not promote a variation in the number of cells.

**Figure 1:**
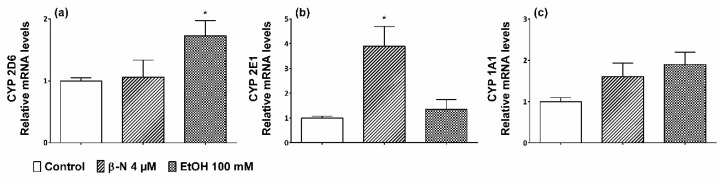
mRNA levels of CYP2D6, 2E1, and 1A1 in SH-SY5Y cells treated with β-naphtoflavone (β-NF) and EtOH. The relative mRNA levels were measured by qRT-PCR for CYP 2D6 (**a**), CYP 2E1 (**b**), and CYP 1A1 (**c**). The results represent the mean ± SEM of at least three different experiments. Each column represents the fold increase calculated after a ΔΔ*C*t analysis of each treatment compared with controls and normalised with rRNA 18S as a housekeeping gene. Controls of each isoform were always taken as 1-fold increase. * *p* < 0.05 vs. control.

In undifferentiated cells, the mRNA levels of CYP2D6 were not significantly affected by β-NF treatment, yet EtOH promoted a significative 1.7-fold increase (Figure 1a). Moreover, CYP2E1 promoted an increase of 4-fold changes after β-NF treatment while EtOH only showed a nonsignificant 1.4-fold change (Figure 1b). None of the treatments statistically increased the mRNA levels of CYP1A1, although β-NF and EtOH showed a 1.6- and 1.9-fold increase, respectively (Figure 1c). CYP3A4 was not analyzed because it was included in the study during the WB analysis and we did not find any change of expression with the treatments.

**Figure 2:**
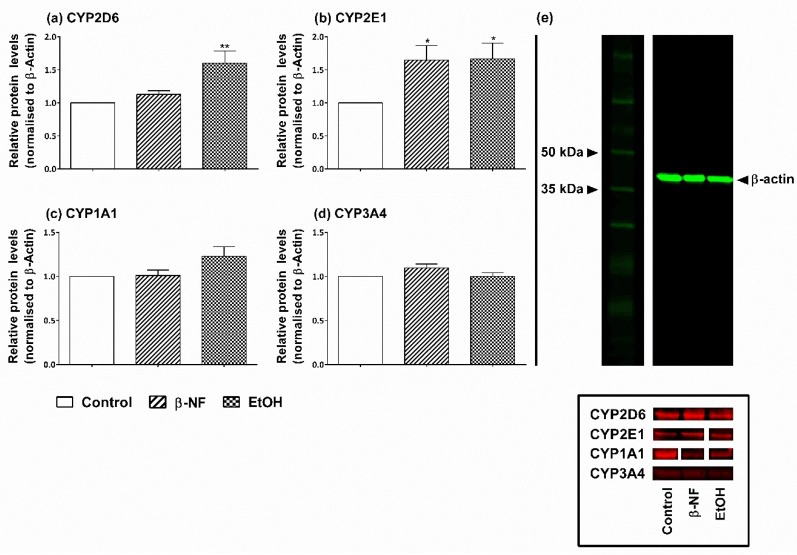
Effect of β-NF and EtOH treatment on cytochrome P450 (CYP) isoforms’ expression in undifferentiated SH-SY5Y cells. (**a**–**d**) The graphs show the relative protein levels quantification by Western blot for the CYP2D6 (**a**), 2E1 (**b**), 1A1 (**c**), and 3A4 (**d**) isoforms after treating cells with the inducers for 48 h (see Section 4). Data was acquired by measuring the fluorescent intensity per pixel on each band. The relative amount of protein normalised with β-actin as a housekeeping protein for each condition was plotted as fold-increase and compared to the control, which was given a value of 1. Columns represent the mean ± SEM of at least three different experiments. Statistical significance was analyzed by one-way ANOVA followed by a Tukey post-test (* *p* < 0.05; ** *p* < 0.01). (**e**) Top panel shows a representative blot of β-actin housekeeping protein detected with secondary antibody Cy3 (green) in control (2nd lane), β-NF (3rd lane) and EtOH (4th lane) treatments. (**e**) Bottom panel shows representative blots of each isoform detected with secondary antibody Cy5 (red) in the mentioned conditions.

Since post-transcriptional mechanisms can lead to different protein expression pattern compared to their respective mRNA levels, and to validate the presence of each isoform in SH-SY5Y cells, a WB analysis was carried out in both undifferentiated and differentiated cells. In the undifferentiated phenotype, the protein levels of CYP2D6 revealed that EtOH was the most potent inducer, showing a 1.6-fold increase compared to control, while β-NF did not significantly affect the expression of this protein (Figure 2a). Moreover, CYP2E1 showed to be inducible by both β-NF and EtOH by about 1.7-fold (Figure 2b). The WB results of CYP1A1 showed the same non-induction pattern by both treatments, as well as for CYP3A4 (Figure 2c,d).

Differentiated cells presented a significant increase of CYP 2D6 protein levels after treatment with either β-NF or EtOH of 1.5- and 1.4-fold increase, respectively (Figure S2a). On the contrary, the treatment with β-NF did not promote the increase in the expression of CYP 2E1, while EtOH significantly increased it (Figure S2b). Neither CYP1A1 nor CYP3A4 were affected by both treatments in differentiated cells (Figure S2c,d).

**Figure 3:**
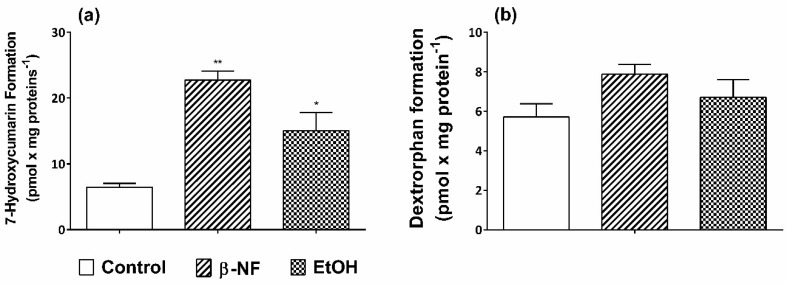
Treatment with β-NF and EtOH increase the metabolic activity of CYP(s) in undifferentiated SH-SY5Y cells. Cells were incubated for 48 h with β-NF and EtOH and were subsequently treated for 24 h with either 7-ethoxycoumarin (50 µM) or dextromethorphan (50 µM). (**a**) 7-Ethoxycoumarin dealkylase activity in SH-SY5Y cells is increased after treatment with inducers. 7-Hydroxycoumarin was determined spectrofluorimetrically by an excitation of 370 nm and emission of 455 nm. (**b**) Dextrorphan formation in β-NF- and EtOH-treated cells. Each column represents the metabolite formation (dextrorphan) and was determined by HPLC–MS analysis. Results are expressed as mean ± SEM of three different cell preparations. * *p* < 0.05; ** *p* < 0.01.

7-Ethoxycoumarin is metabolized by many CYP isoforms involved in xenobiotic metabolism [62]. For this reason, it has been used as a prototypic substrate to monitor CYPs activity in both hepatic and extrahepatic tissues. The treatment of SH-SY5Y cells with β-NF and EtOH promoted a significant increase in CYPs activity. In fact, the amount of 7-hydroxycoumarin recovery after 48 h incubation with the inducers was greater and statistically different than the control values (Figure 3a). The formation rate of 7-hydroxycoumarin showed a 3.51- and 2.32-fold increase by β-NF and EtOH treatments, respectively. The dextromethorphan is metabolized mainly by CYP2D6, which promotes the formation of dextrorphan by an *O*-demethylation reaction [63]. The HPLC–MS analysis revealed, after 24 h incubation with dextromethorphan, a peak at *m*/*z* value corresponding to [M − 15]^+^ and identified by means of Mass-MetaSite^®^, a computer-assisted method for the interpretation of LC–MSMS data, as the demethylated metabolite, dextrorphan. Other peaks, characterized by a very low intensity at [M + 16]^+^ *m*/*z* were observed. As shown in Figure 3b, an increase of dextrorphan formation, although not statistically significant, was observed with both treatments (+38% after β-NF and +19% after EtOH).

### 2.2. CYP2D6 Localizes in Mitochondria

**Figure 4:**
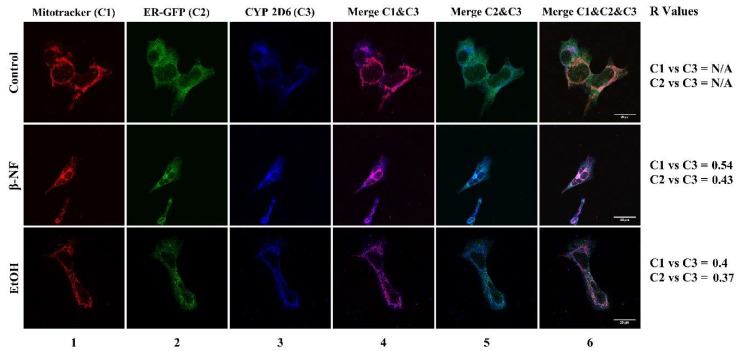
Immunostaining of CYP2D6 in SH-SY5Y cells after β-NF and EtOH treatment. Representative immunofluorescence images of each experimental condition. Column 1 represents mitochondrial stain (red), column 2 represents ER-Green Fluorescent Protein (GFP) stain (green), and column 3 reports the CYP2D6 staining (blue). Column 4 represents the merge between CYP2D6 and mitochondria channel, column 5 represents the merge between CYP2D6 and ER-GFP, and column 6 represents the merge of the three channels. *R* Values: Pearson’s correlation coefficient. N/A: Not determined. Scale bars: 20 µm (control and EtOH) and 40 µm (β-NF).

Following the same induction protocol, cells were treated with the inducers and the four isoforms were fluorescently marked in order to study their localization in the undifferentiated cells. Interestingly, the expression of CYP2D6 after treatments correlated with mitochondria, while it presented less localization in ER (Figure 4). The resulting *R* values for mitochondrial localization were 0.54 and 0.4 for β-NF and EtOH, respectively. Localization between CYP2D6 and ER was lower, as observed by their *R* Pearson coefficients (β-NF, 0.43; EtOH, 0.37). It must be underlined that due to low fluorescent intensity in control conditions, the colocalization plugin was not able to correlate the CYP channel with either mitochondria or ER.

**Figure 5:**
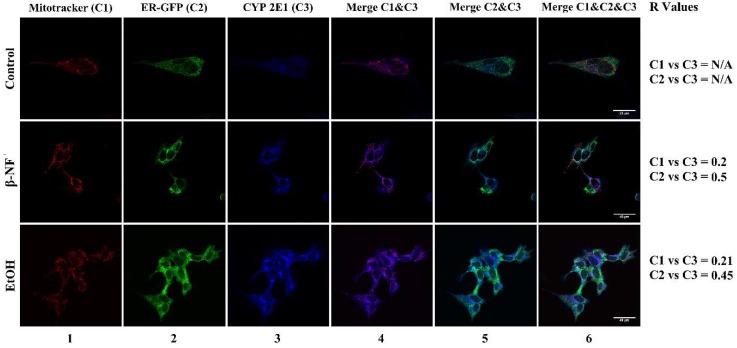
Immunostaining of CYP2E1 in SH-SY5Y cells after β-NF and EtOH treatment. Representative immunofluorescence images of each experimental condition. Column 1 represents mitochondrial stain (red), column 2 represents ER-Green Fluorescent Protein (GFP) stain (green), and column 3 reports the CYP2E1 staining (blue). Column 4 represents the merge between CYP2E1 and mitochondria channel, column 5 represents the merge between CYP2E1 and ER-GFP, and column 6 represents the merge of the three channels. *R* Values: Pearson’s correlation coefficient. N/A: Not determined. Scale bars: 20 µm (control), 40 µm (β-NF and EtOH).

The analysis of the CYP2E1 fluorescence revealed that this isoform was localized with ER and not with mitochondria (Figure 5). The *R* values for mitochondrial localization were 0.20 and 0.21 for β-NF and EtOH treatments, respectively; while localization in ER showed *R* values of 0.50 and 0.45 for β-NF and EtOH treatments, respectively. Similar to CYP2D6, the *R* Pearson coefficient was not able to be calculated in control conditions due to the low fluorescent intensity.

**Figure 6:**
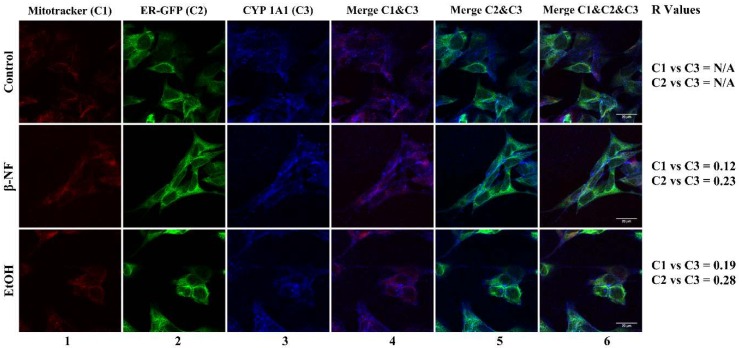
Immunostaining of CYP1A1 in SH-SY5Y cells after β-NF and EtOH treatment. Representative immunofluorescence images of each experimental condition. Column 1 represents mitochondrial stain (red), column 2 represents ER-Green Fluorescent Protein (GFP) stain (green), and column 3 reports the CYP1A1 staining (blue). Column 4 represents the merge between CYP1A1 and mitochondria channel, column 5 represents the merge between CYP1A1 and ER-GFP, and column 6 represents the merge of the three channels. *R* Values: Pearson’s correlation coefficient. N/A: Not determined. Scale bar: 20 µm.

Fluorescent images of CYP1A1 revealed no preferential localization of this isoform at mitochondria or ER, yet the *R* values showed to be slightly higher in ER (Figure 6). The *R* values of mitochondrial localization in β-NF and EtOH treatments were 0.12 and 0.19, respectively; while the *R* values of ER localization were 0.23 and 0.28, respectively. Similar to CYP2D6 and 2E1, the low expression of CYP1A1 in the control condition did not allow us to calculate the *R* Pearson coefficient.

**Figure 7:**
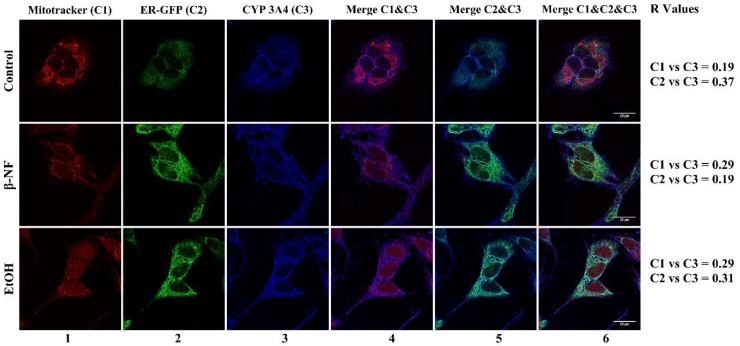
Immunostaining of CYP3A4 in SH-SY5Y cells after β-NF and EtOH treatment. Representative immunofluorescence images of each experimental condition. Column 1 represents mitochondrial stain (red), column 2 represents ER-Green Fluorescent Protein (GFP) stain (green), and column 3 reports the CYP3A4 staining (blue). Column 4 represents the merge between CYP3A4 and mitochondria channel, column 5 represents the merge between CYP3A4 and ER-GFP, and column 6 represents the merge of the three channels. *R* Values: Pearson’s correlation coefficient. Scale bar: 20 µm.

We also observed that the fluorescent images of CYP3A4 did not report a preferential localization between mitochondria and ER (Figure 7). The *R* value in mitochondria of both β-NF and EtOH treatments were 0.29, while the values obtained in ER were 0.19 and 0.31, respectively.

### 2.3. Protective Effect of CYPs

**Figure 8:**
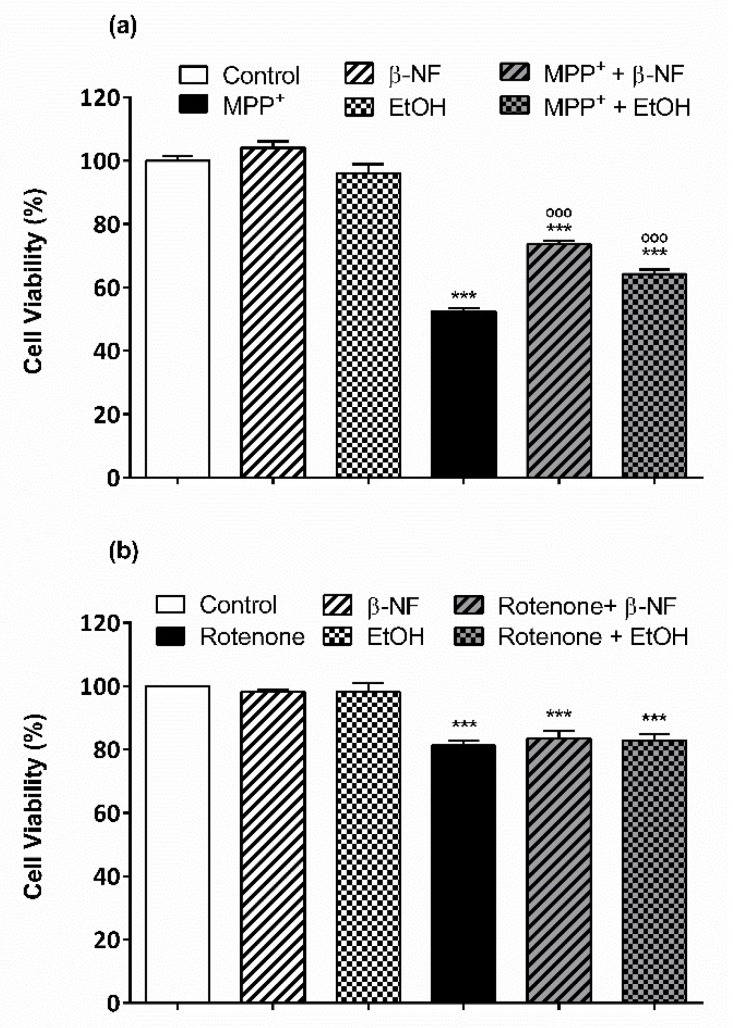
β-NF and EtOH treatments partially protect SH-SY5Y cells towards MPP^+^ exposure but not against rotenone. SH-SY5Y cells were pretreated with β-NF or EtOH for 24 h and with 600 µM MPP^+^ (**a**) or 0.2 µM rotenone (**b**) for the following 24 h. No treatment was performed in control cells. Viability was tested by tetrazolium (MTT) assay as described in materials and methods. Each column represents the mean ± SEM of at least three independent experiments. Data was normalized by taking control values as 100% of metabolic activity, and then compared for significance to control samples (*) and to MPP^+^ or rotenone controls (°). Statistical significance was analyzed by one-way ANOVA followed by a Tukey post-test. *** *p* < 0.001 with respect to controls; °°° *p* < 0.001 with respect to MPP^+^ or rotenone.

The role of CYP2D6 and 2E1 in neurodegeneration is important for developing new therapeutic approaches. We evaluated the effect that β-NF and EtOH treatments have upon exposure to either MPP^+^ (600 µM) or rotenone (0.2 mM) in SH-SY5Y cells. Both compounds were shown to have a partial protective effect against MPP^+^ but not towards rotenone. In fact, both inducers were able to promote a slight but significant increase in cell viability compared to MPP^+^ controls (Figure 8a). This toxin decreased cell viability by 50% compared to control cells. β-NF resulted in an increase of cell viability of 22% compared to MPP^+^ controls, while EtOH promoted an increase of 18%. On the contrary, the cell viability after rotenone exposure resulted in a nonsignificant decrease of 5% and an increase of 7% in β-NF and EtOH treatments, respectively (Figure 8b).

## 3. Discussion

CYP isoforms are involved in several metabolic processes that are important for cell homeostasis and cell defense against xenobiotic insult. The level of these isozymes is lower in the brain compared to the liver. However, they can play a role in the (in)activation of neurotoxins, as well as affect the therapeutic properties of drugs. In order to clarify its conceivable role in activation/deactivation of neurotoxins, we have investigated the possibility to induce some isoforms *in vitro* in neuroblastoma SH-SY5Y cells, a model commonly used for research in neurodegenerative diseases. Our results demonstrate that CYP2D6 and 2E1 can be increased in terms of mRNA, proteins, and activities by incubating undifferentiated SH-SY5Y cells with two well-known inducer compounds in the liver, β-NF and EtOH. β-NF is known to induce CYP isoforms belonging to the family 1A and 1B, while EtOH induces the expression of CYP2E1 isoform. Moreover, different studies suggested that, inconsistent with what has been observed in the liver, CYP2D6 is inducible in the brain and in SH-SY5Y [30,35]. Therefore, we have also investigated the effects of β-NF and EtOH on CYP2D6.

Under our experimental conditions, CYP2D6 seems to be inducible by EtOH in terms of mRNA and protein levels, and not by β-NF, while in differentiated cells, both β-NF and EtOH were able to increase the protein levels. Moreover, CYP2E1 mRNA levels were statistically increased by β-NF and not by EtOH. However, both treatments were able to increase the CYP2E1 proteins as observed by WB analysis in undifferentiated cells, while only EtOH promoted this expression in the differentiated phenotype. On the other hand, CYP1A1 was unexpectedly not induced by β-NF. Taken together, these results indicate that the regulation pathways of CYPs in SH-SY5Y cells are different compared to those observed in liver in vivo and in vitro conditions. Our results are in agreement with Imran et al. [64], who showed that 2,3,7,8-tetrachlordibenzo-p-dioxin (TCDD), one of the most potent inducers of CYP1 family via AhR, is able to increase the mRNA levels of these CYP isoforms only at 10 nM in SH-SY5Y cells, while 2.5 and 5 nM did not have this effect. On the contrary, in differentiated SH-SY5Y cells, this compound was able to increase the expression of CYP1A1 at 2.5 and 5 nM. This could be due to the absence of AhR in SH-SY5Y. β-NF, as TCDD and other aromatic hydrocarbons, interacts with the same pathway in the regulation of CYPs in the liver. Therefore, evidence of the lack or lower AhR expression in SH-SY5Y cells could explain the failure of β-NF in regulating the expression of CYP(s) in SH-SY5Y cells. On the other hand, the EtOH induction of CYP2D6 observed in these results is in agreement with other studies, where an in vitro induction of this isoform by the same compound has been reported [65]. Furthermore, the in vivo treatment of African green monkeys with nicotine also demonstrated that CYP2D6 can be inducible in brain while remaining unchanged in the liver [41]. The evidence of the induction of CYP2D6 in SH-SY5Y cells, in conflict with that observed in hepatocytes, suggests that this isoform has a different regulation in the neuroblastoma cell line. Recently, Zhang, et al. [66] suggested the involvement of PPARs in the regulation of CYP2D6 in brain and SH-SY5Y cells. Conversely, the treatment with EtOH did not show a significant increase of the CYP2E1 mRNA levels, while in WB analysis we observed a statistical increase of protein levels. These results are justified by the evidence that EtOH promotes CYP2E1 protein stabilization, rather than regulation via receptor activation [67]. Moreover, the present results agree with those obtained by other research groups [58,68,69]. CYP2E1 induction was also observed in vivo, where rats treated with EtOH and nicotine had increased brain levels of this isoform [70].

As shown in WB experiments, the expression of CYP2D6 and 2E1 proteins can be increased by at least one compound used in this study in both undifferentiated and differentiated phenotypes. In general terms, these results support the inducibility of CYP2D6 and 2E1 in our in vitro model, in contrast to other isoforms such as CYP1A1 and 3A4. Furthermore, the WB experiments performed in differentiated cells provide a more realistic view of the CNS, taken into account the different expression patterns of CYP isoforms in brain cells/areas [71]. The role of CYP2D6 in PD is still under debate. In fact, a pharmacogenetic study of 70 individuals with PD showed that a lower metabolic rate of CYP2D6 was associated with a higher risk of developing this disease [72]. However, other groups state that the association between PD and mutations of CYP2D6 is most likely the result of interactions between multiple genetic and environmental factors [73]. It is also important to underline the contrasting results in the literature on the role of CYP2E1 in the brain. A protective role of this isoform against MPTP has been suggested [74]. However, in another study by the same group, the knockout mice of *CYP2E1*^−/−^ were shown to have a lower degeneration of dopaminergic neurons after acute administration of this toxin [75]. On the other hand, a study of six PD patients showed a lower DNA methylation at the promoter region of *CYP2E1* gene in both cortex and putamen, and an increase of mRNA levels in cortex, suggesting that epigenetic variations of this isoform may contribute to PD susceptibility, but they did not study the correlation between mRNA levels and proteins or enzymatic activity [76].

In order to study the metabolic capability of SH-SY5Y cells after the induction of CYP isoforms, we used 7-ethoxycoumarin as substrate for a broad number of CYP isozymes [62]. Several studies indicated that, in human liver microsomes, CYP1A1, 1A2, 2E1, 2A6, and 2B6 are the major isoforms involved in ethoxycumarin *O*-dealkylation reaction, although other isoforms such as 1B1, 2C, and 2D6 can play a minor role [77]. However, CYP2D6 is one of the most expressed isoforms in dopaminergic neurons [2], and therefore, the evidence that our treatments in SH-SY5Y cells increased 7-hydroxycumarin formation clearly indicated that the induction of CYP(s) proteins also corresponded to an increase in the CYP-dependent metabolism. The activity of 2D6 was also tested using dextromethorphan as the marker substrate. The results demonstrate that cells are able to promote the CYP2D6-dependent metabolism of the compound although its induction does not lead to a statistically significant increase compared to control. This can be explained by the fact that dextromethorphan is only metabolized by CYP2D6, unlike 7-ethoxycoumarin, and its low concentration in our cellular preparations, even in the case of induction, is not sufficient to increase the formation of dextrorphane in a statistically significant manner.

Another question to address is the intracellular expression of these isoforms in SH-SY5Y cells. It is established that, in general, hepatic CYPs are located in the ER. However, it has been shown that some isoforms can be present at the mitochondrial level in the brain [78,79]. The localization of CYP in mitochondria can contribute to the bioactivation/detoxification processes of neurotoxins. Indeed, xenobiotics involved in neurodegeneration, such as MPP^+^, paraquat, and rotenone, target the mitochondria, leading to the impairment of their functions. Our study showed by confocal microscopy analysis that CYP2D6 in treated cells also localized at mitochondria, and at a minor level, at ER. The intracellular location of this isoform is still a matter of debate, as no studies have confirmed a mitochondrial localization. Targeting this organelle is a key feature for the implications of CYP2D6 in neurodegeneration. Some polymorphisms have been found in liver mitochondria, and, in other in vitro studies, neurons have been transduced to overexpress a mitochondrially-targeted CYP2D6 [4,80,81,82]. This experimental evidence may represent another important perspective of the role of CYP2D6 in detoxification processes, since this isoform is proposed to be neuroprotective against xenobiotics that target the mitochondria, such as the MPP^+^ [35]. Like MPTP, other toxins (i.e., rotenone and paraquat) promote neurodegeneration by impairing the mitochondrial functions, and, for these reasons, mitochondria represent an important target for developing new useful drugs in neurodegenerative diseases [73].

In contrast, CYP2E1 appeared to partially localize in the ER, as it has been reported in other cell types and tissues [3,53,83]. Recently, in other neuroblastoma cell lines, this isoform presented very low expression levels [84]. With the difficulty of the quantitative and qualitative analysis of the induction, this could explain why the fluorescence in some tested conditions did not reach the threshold required for the calculation of the R coefficient in our experiments. Moreover, other groups have indicated the possibility that CYP2E1, and other isoforms, may target the mitochondria in other in vitro and in vivo models [3,5,6,83,85]. However, the intracellular location of CYP2E1 is still a subject of debate. It has been proposed that in rat neurons, this isoform is located at a consistent distance from the nucleus, possibly in the mitochondria, but this can vary between cell types [85]. The localization images of CYP1A1 and 3A4 showed no preferential localization of these isoforms for either mitochondria or ER, as also shown by other groups in several models [3,6]. However, the expression of the four isoforms in general can vary depending on the brain area and cellular type, and the only expression of CYP2D6 at mitochondrial level highlights the importance of this isoform only in neuroblastoma SH-SY5Y cells. Therefore, other CYP isoforms can have an important role in xenobiotic metabolism in other brain regions, and at the same time, they do not have to necessarily be associate with positive effects [9,71].

Finally, our results in cell viability suggest that the induction of CYP2D6 and 2E1 might have a protective role against the toxicity promoted by MPP^+^. This toxin can be metabolized by the CYP system as already reported [35,86]. Indeed, the partial neuroprotection showed by β-NF and EtOH treatments agree with other MTT experiments, where the inhibition of CYP2D6 increased the toxic effect of MPP^+^ in SH-SY5Y cells [35]. On the other hand, CYP2E1 has been proposed to be involved in the intracellular accumulation of this toxin rather than in the metabolism itself [86]. Thus, the increase of these two isoforms showed in this work, and by other groups, supports the protective effect of this metabolic system against the toxicity of MPP^+^ [58,65,80,87]. Cells exposed to rotenone after incubation with inducers were not able to avoid the toxic effects. However, the toxicity promoted by similar concentrations of rotenone can be reduced in SH-SY5Y cells in the presence of natural flavonoids, promoting neuroprotection [88]. Since CYP is not able to metabolize this compound, we confirmed that the effects observed were not produced by the inducers itself, but by the promotion of the CYP system. However, other experiments are necessary to confirm the partial neuroprotection promoted by β-NF and EtOH in our in vitro model. Other groups have shown that the active site of CYP2D6 is large enough to allow MPP^+^ to enter into it, suggesting that the metabolism of this toxin can be carried out by this isoform [89]. Since CYP2D6 and MPP^+^ target mitochondria, we expect that the toxic effect and the protection by CYPs observed here will affect mitochondrial function. Therefore, viability assays at cellular and mitochondrial level will address this issue.

## 4. Materials and Methods

### 4.1. Cell Culture

The human neuroblastoma SH-SY5Y cells (ECACC, #94030304) were purchased from Sigma-Aldrich (Milan, Italy). SH-SY5Y cells were cultured in polystyrene-coated flasks in RPMI-1640 medium supplemented with 10% fetal bovine serum (FBS), 100 U/mL of penicillin, and 100 mg/L streptomycin (Sigma-Aldrich). Cultures were maintained at 37 °C in 5% CO_2_ and medium was changed twice per week. When flasks were at about 80% confluency, the culture was passaged at a ratio of 1:10. Only cultures between passages 3 and 13 were used.

In separate experiment, differentiation to a more dopaminergic phenotype was carried out using the previously reported protocol [90,91]. In brief, when cells were at 75% confluency, medium was changed for new fresh 1% FBS medium and 10 µM retinoic acid. After three days, medium was changed for fresh 1% FBS medium and 80 nM phorbol ester, and cells were incubated for an additional three days.

### 4.2. Quantitative Real-Time PCR

The quantification of relative mRNA levels for the isoforms CYP1A1, 2D6 and 2E1 was carried out after incubation of SH-SY5Y cells for 48 h with β-NF (4 µM), dissolved in dimethyl sulfoxide (DMSO; 0.1% maximum final concentration used, *v*/*v*), and EtOH (100 mM), dissolved in water. In brief, a day after seeding (10^6^ cells in 75 cm^2^ flasks), cells were treated with the inducers for 48 h. RNA was purified with TRIzol reagent (Life Technologies; Milan, Italy) according to the manufacturer indications and eluted in RNase-free water. Only the samples with an OD_260/280_ ratio between 1.9 and 2.1 were selected. From each sample, 1 µg of total RNA was used for reverse transcription with the High-Capacity cDNA Reverse Transcription Kit (Life Technologies) according to the manufacturer indications.

The resulting cDNA samples were used for PCR amplification in a StepOne™ Real-Time PCR System (Life Technologies) using the TaqMan chemistry. The following probes for CYP isoforms were used, CYP1A1 (Life Technologies, Assay ID Hs01054797_g1, # 4331182), CYP2D6 (Life Technologies, Assay ID Hs03043790_g1, # 4331182), and CYP2E1 (Life Technologies, Assay ID Hs00559368_m1, # 4453320). Probes had the 5′-end labeled with a FAM™ dye as a reporter and the 3′-end labeled with an MGB molecule as a quencher.

The average threshold cycle (C_t_) of the technical duplicates of each PCR reaction was used for quantification and was applied to the 2^−ΔΔ*C*t^ method as described by Livak and Schmittgen [92] and by Pfaffl [93]. The results were normalized using rRNA 18S as a housekeeping gene. The probe for the rRNA 18S gene (Life Technologies, Assay ID Hs03928985_g1, # 4331182) was labeled at the 5′-end with a VIC^®^ dye as a reporter and with an MGB molecule at the 3′-end as a quencher. The housekeeping gene was amplified with the isoforms in the same reactions under the same experimental conditions. Normalized results were shown as fold change values compared with the control untreated cultures, which were taken as one-fold value.

### 4.3. Western Blot and Relative Protein Level Quantification

The analysis of protein levels was performed for isoforms CYP1A1, 2D6, 2E1, and 3A4 in both undifferentiated and differentiated SH-SY5Y cells. The protein extraction protocol was performed after 48 h incubation with inducers as already described above for qRT-PCR assays. Cells were scrapped in RIPA lysis buffer with a protease and phosphatase inhibitor cocktail (Sigma-Aldrich) and proteins were isolated by centrifugation. To determine the protein concentration, a Bradford reagent (Sigma-Aldrich) was used according to manufacturer indications.

Western blotting with endogenous protein normalization was performed with an Amersham™ WB System (GE Healthcare; Milan, Italy). In brief, 20 µg/sample was loaded in a 13.5% gel card and separated proteins were then transferred to a polyvinylidene difluoride (PVDF) card and membranes were blocked with a dry milk solution (3% *w*/*v*) for 10 min. They were incubated with one of the following primary antibodies overnight, anti-CYP1A1 (Life Technologies, # PA515213, 1:1000, polyclonal rabbit), anti-CYP2D6 (Life Technologies, # PA535148, 1:1000, polyclonal rabbit), anti-CYP2E1 (Life Technologies, # PA535351, 1:1000, polyclonal rabbit), or anti-CYP3A4 (Life Technologies, # PA514896, 1:1000, polyclonal rabbit) mixed with anti-β-actin primary antibody (Life Technologies, # PA516914, 1:1000, polyclonal mouse) as an endogenous housekeeping protein. Membranes were then incubated for two hours with secondary antibodies: goat anti-rabbit Cy™5 (GE Healthcare, # 29038278, 1:1000) and goat anti-mouse Cy™3 (GE Healthcare, # 29038275, 1:1000). Fluorescence membrane scan and analysis of pixel intensity for each band was carried out with the Amersham™ WB software (GE Healthcare; Version 1.0.33.1695). Each sample value was plotted as a normalized ratio related to control values and expressed as fold change (controls were taken as 1-fold).

### 4.4. Cytochrome P450-Dependent Activity Assay

The CYPs activities were assayed using 7-ethoxycoumarin (Sigma-Aldrich) as substrate of multiple CYP isoforms [62] or dextromethorphan (Sigma-Aldrich) as CYP2D6 marker substrate. The 7-hydroxycoumarin production (CYPs-dependent metabolite) was analyzed fluorometrically as previously reported [94], while the dextromethorphan metabolism was analyzed by mass spectrometry using an Agilent 6540 UHD Accurate-Mass Q-TOF LC/MS spectrometer operated in positive electrospray mode, reported elsewhere [95]. In brief, SH-SY5Y cells were treated with the inducers for 48 h. After incubation, the medium was changed and cells were incubated for another 24 h in a culture medium containing 7-ethoxycoumarin or dextromethorphan at a final concentration of 50 µM. Then, cells and medium were separately collected and the reaction was cooled down and quenched with acetonitrile (Sigma-Aldrich, 0.5 mL). After centrifugation (3000× *g*, 10 mins), the supernatant was discarded and the pellet was dried and resuspended in 500 µL of distilled water. In the case of the dextromethorphan metabolism assay the pellets were dissolved in 100 µL acetonitrile (Sigma-Aldrich). The calibration curves were obtained using increasing concentrations of the analyte (0–0.1 µM) either for 7-hydroxycoumarin and dextrorphan. The enzyme activities were expressed as pmol metabolite per mg of protein in 24 h.

### 4.5. Confocal Microscopy

A staining approach to evaluate the CYP location in SH-SY5Y was carried out by confocal imaging. Cells were plated on coverslips in 6-well plates at 1 × 10^5^ cells/well. After 24 h, cells were treated with either 4 µM β-NF or 100 mM EtOH for two days. Twenty-four hours prior to fixation, 5 µL of endoplasmic-reticulum-green fluorescent protein (Life Technologies; ER-GFP) reagent was added to each well and cells were incubated overnight. Cells were then treated with MitoTracker^®^ Red CMXRos (Life Technologies) at a concentration of 50 nM for 30 min. Fixation was carried out with a 4% paraformaldehyde solution for 10 min at room temperature followed by a permeabilization with 0.1% Triton X-100 solution (Sigma-Aldrich) for 2 min. Cells were then blocked with a 1% bovine serum albumin solution for 1 h at room temperature. Samples were incubated overnight at 4 °C with the same primary antibodies used for WB in blocking solution, then incubated with secondary antibody goat anti-rabbit Cy™5 in blocking solution for two hours at 4 °C.

Image acquisition was performed using a Leica TCS SP8 STED microscope (Leica microsystems, Ashbourne, Ireland) equipped with an HC PL APO CS2 63x/1.40 OIL objective. Images were analysed with LAS X 2.0.1.14392 (Leica microsystems, Ashbourne, Ireland) and “Coloc 2” plugin in Fiji’s software (Madison, WI, USA).

### 4.6. MTT Assay

Cells were plated in 96-well plates at a concentration of 1.6 × 10^4^ cells/well and treated with β-NF and EtOH separately for 24 h. After incubation, cells were washed and treated with either MPP^+^ (Sigma-Aldrich, 0.6 mM), dissolved in water or rotenone (Sigma-Aldrich, 0.2 µM), then dissolved in DMSO (0.1% maximum final concentration used, *v*/*v*). The MTT assay was carried out as reported by Sylvester [96] with some modifications. In brief, medium was changed to new complete medium containing 0.5 mg/mL of MTT (Sigma-Aldrich) dissolved in complete medium and cells were incubated for 90 min at 37 °C and 5% CO_2_. The medium was removed and the resulting formazan was dissolved in DMSO. Plates were shaken for 10 min and absorbance was recorded at 540 nm with a plate reader (Multiskan GO, SkanIT software version 3.2; Thermo Fisher Scientific, Milan, Italy). The mean values of each treatment from independent experiments were normalized to percentages related to mean control values, which were taken as 100% viability.

### 4.7. Statistical Analysis

Data from WB, enzymatic activity and MTT assays were analysed for statistical significance by one-way ANOVA followed by Tukey’s post-hoc test. Data from qRT-PCR were analysed for statistical significance by Student’s unpaired *t*-test according to Yuan et al. [97]. The statistical analysis was performed with GraphPad Prism Software, Version 6.01. For each experiment, at least three biological repetitions were performed. The colocalization tests of confocal microscopy pictures were carried out by “Coloc 2” plugin from Fiji’s Software. R Pearson values indicate the level of colocalization. *R* = 1 correspond to a perfect colocalization and *R* = 0 to no colocalization.

## 5. Conclusions

In conclusion, the present study demonstrates that in SH-SY5Y cells, CYP2D6 can be induced by EtOH and CYP2E1 by β-NF and EtOH. We also propose a primary role of CYP2D6 toward the metabolism of compounds related to PD due its presence in mitochondria and suggest that SH-SY5Y cells can be a useful in vitro experimental method to clarify the overall role of CYPs at neuronal level.

## Supplementary Materials

Supplementary materials can be found at http://www.mdpi.com/1422-0067/19/11/3369/s1.

## Abbreviations

AhR	Aryl hydrocarbon receptor
*C*_t_	Threshold cycle
CYP	Cytochrome P450
DMSO	Dimethyl sulfoxide
ER	Endoplasmic reticulum
ER-GFP	Endoplasmic reticulum-green fluorescent protein
EtOH	Ethanol
FBS	Fetal bovine serum
MPP^+^	1-methyl-4-phenylpyridinium iodide
MPTP	1-methyl-4-phenyl-1,2,3,6-tetrahydropyridine
qRT-PCR	Real time quantitative-polymerase chain reaction
WB	Western blot
β-NF	β-naphtoflavone
