# Supplementary material for: β-Naphtoflavone and Ethanol Induce Cytochrome P450 and Protect towards MPP+ Toxicity in Human Neuroblastoma SH-SY5Y Cells
Source: Int J Mol Sci. 2018 Oct 28;19(11):3369. doi: 10.3390/ijms19113369 (PMC6274691; doi:10.3390/ijms19113369)
Supplement: Supplementary file 1 [file ijms-19-03369-s001.pdf]

Supplementary: β-Naphtoflavone and Ethanol Induce Cytochrome P450 and Protect towards MPP^+ Toxicity in Human Neuroblastoma SH-SY5Y Cells

Jesus Fernandez-Abascal [1,*,†], Mariantonia Ripullone [1], Aurora Valeri [2], Cosima Leone [1] and Massimo Valoti [1]

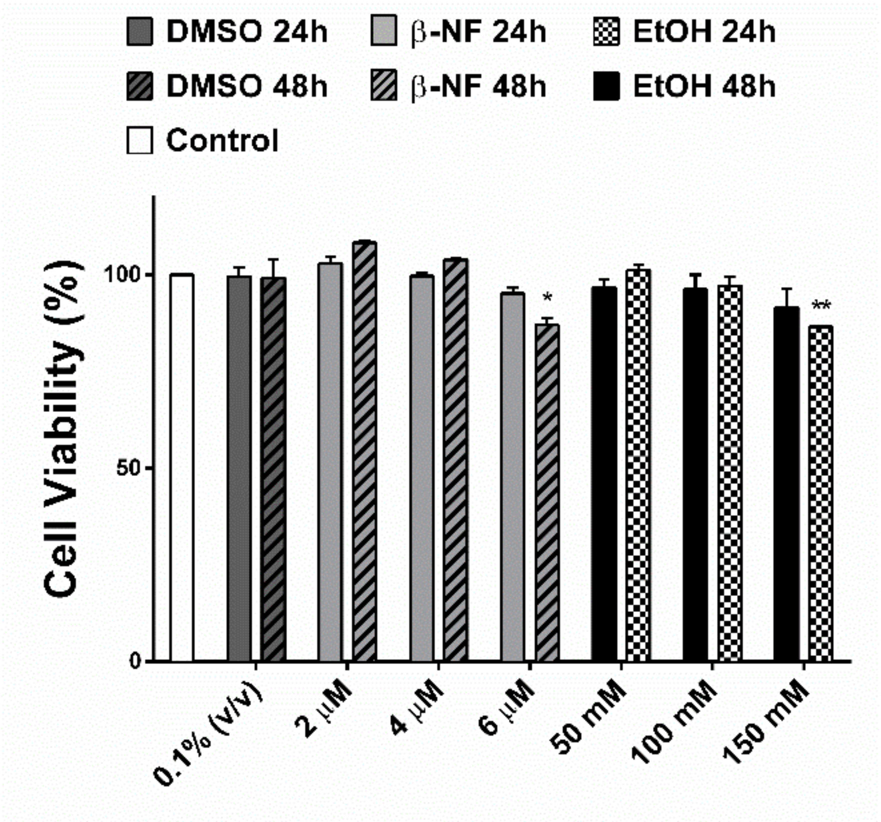

Figure S1. Time and dose toxicity of β-NF and EtOH treatments. Undifferentiated SH-SY5Y cells were treated with β-NF (2, 4 and 6 μM) or EtOH (50, 100 and 150 mM) for 24 and 48 hours. No treatment was performed in control cells. Viability was tested by MTT assay as described in materials and methods. Each column represents the mean ± SEM of at least three independent experiments. Data was normalized by taking control values as 100% of metabolic activity, and then compared for significance to control samples (*). Statistical significance was analyzed by one-way ANOVA followed by a Tukey post-test (*$p<0.05$; **$p<0.01$).

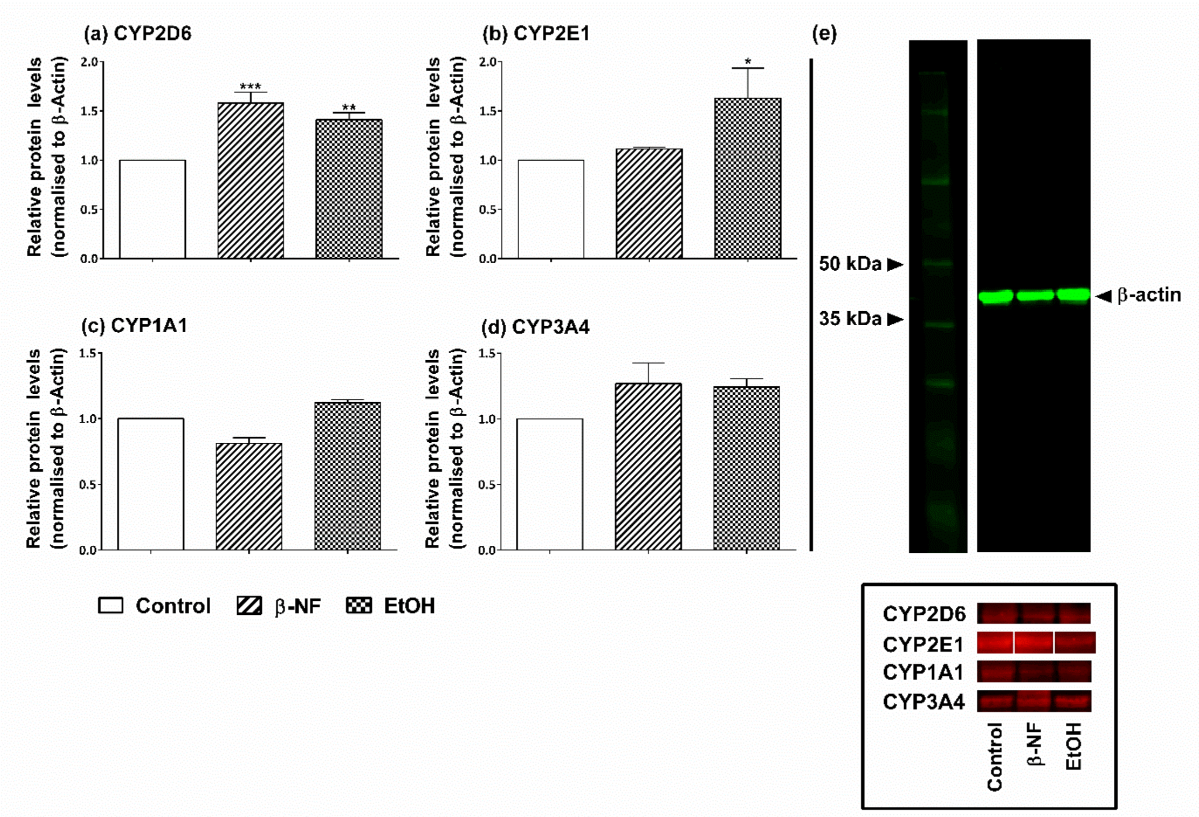

Figure S2. Effect of β-NF and EtOH treatment on CYP isoforms' expression in differentiated SH-SY5Y cells. (**a-d**) The graphs show the relative protein levels quantification by western blot for the CYP 2D6 (a), 2E1 (b), 1A1 (c) and 3A4 (d) isoforms after treating cells with the inducers for 48h (see methods). Data was acquired by measuring the fluorescent intensity per pixel on each band. The relative amount of protein normalised with β-actin as a housekeeping protein for each condition was plotted as fold-increase and compared to the control, which was given a value of 1. Columns represent the mean ± SEM of at least three different experiments. Statistical significance was analyzed by one-way ANOVA followed by a Tukey post-test (*$p<0.05$; **$p<0.01$; ***$p<0.001$). (**e**) Top panel shows a representative blot of β-actin housekeeping protein detected with secondary antibody Cy3 (green) in control (2nd lane), β-NF (3rd lane) and EtOH (4th lane) treatments. (**e**) Bottom panel shows representative blots of each isoform detected with secondary antibody Cy5 (red) in the mentioned conditions.
